# Momentary within-subject associations of affective states and physical behavior are moderated by weather conditions in real life: an ambulatory assessment study

**DOI:** 10.1186/s12966-023-01507-0

**Published:** 2023-09-30

**Authors:** Irina Timm, Markus Reichert, Ulrich W. Ebner-Priemer, Marco Giurgiu

**Affiliations:** 1 Mental mHealth Lab, Institute of Sports and Sports Science, Karlsruhe Institute of Technology, Hertzstr. 16, Karlsruhe, 76187 Germany; 2 Department of eHealth and Sports Analytics, Faculty of Sport Science, Ruhr University Bochum, Gesundheitscampus-Nord 10, Bochum, 44801 Germany; 3 Department of Psychiatry and Psychotherapy, Central Institute of Mental Health, Medical Faculty Mannheim/Heidelberg University, J5, 68159 Mannheim, Germany

**Keywords:** Physical activity, Sedentary behavior, Mood, Context, Temperature, Precipitation, Ecological momentary assessment

## Abstract

**Background:**

Physical behavior (PB) is a key lifestyle factor in regulating and preventing diseases across the lifespan. Researchers identified affective, cognitive, and contextual factors like weather conditions, as significant contributors in determining if individuals are physically active. However, there is scarce empirical evidence about potential associations between PB and affective states influenced by weather conditions in daily life. Therefore, we explored if weather conditions moderated the within-subject association between momentary affective states and subsequent PB.

**Methods:**

Utilizing ambulatory assessment, 79 participants completed electronic diaries about their affective states (i.e., valence, energetic arousal, and calmness) up to six times a day over five days, and their PB (i.e., physical activity and sedentariness) was simultaneously recorded via accelerometers. Weather conditions (i.e., temperature and precipitation) recorded near participants’ locations served as moderators in the multilevel analyses.

**Results:**

We confirmed earlier findings associating affective states with PB. Increased valence and energetic arousal were positively associated with physical activity (β = 0.007; *p* < .001), whereas calmness predicted lower levels of physical activity (β = -0.006; *p* < .001). Higher levels of calmness showed a positive association with sedentary behavior (β = 0.054; *p* = .003). In addition, we revealed a significant positive association between temperature, as a momentary weather condition, and physical activity (β = 0.025; *p* = .015). Furthermore, we showed that the association of affective states and physical activity was moderated by temperature. Higher temperatures enhanced the positive effects of valence on physical activity (β = .001, *p* = .023) and attenuated the negative effects of calmness on physical activity (β = .001, *p* = .021). Moreover, higher temperatures enhanced the positive effects of valence on reduced sedentary behavior (β = -0.011, *p* = .043).

**Conclusions:**

Temperature alterations appeared to have an impact on subsequent physical activity. Furthermore, temperature alterations moderated the influence of affective states on conducted physical activity. This might offer the opportunity for just-in-time adaptive interventions to intervene in individually appropriate environmental conditions for promoting physical activity.

**Supplementary Information:**

The online version contains supplementary material available at 10.1186/s12966-023-01507-0.

## Background

There is compelling evidence that physical activity (PA) has beneficial consequences for both somatic and mental health [1, 2]. However, according to the WHO, 28% of the adult population is not active enough, i.e., does not comply with the activity guidelines of at least 150–300 min of moderate-intensity aerobic PA per week [3, 4]. Based on the definition by Bussmann and van den Berg-Emons, the construct of physical behavior (PB) includes both PA and sedentary behavior (SB) [5]. PA is defined by skeletal muscle effort [6] leading to an increase in energy expenditure and is usually unplanned and unstructured in daily life, e.g., going for a walk or working in the garden. SB is characterized by an energy expenditure lower than 1.5 metabolic equivalents, e.g., while in a sitting or reclined position (excluding sleep) [7]. Being physically inactive, e.g., in terms of prolonged SB, is associated with diabetes, hypertension, obesity, and depression [8], and is related to an increased risk of mortality [9].

The relevance of PB and affective well-being [10] associations for research on behavioral processes underlying entrance to or maintenance of a physically active lifestyle becomes obvious in this field progressing towards a comprehensive approach. Socio-ecological frameworks postulate that individuals are embedded within larger social systems [11], indicating that behavioral, psychosocial, as well as environmental factors contribute to different responses in maintaining or being physically active [12], and can significantly impact physical behavior choices. In this context, affective well-being is defined as an elementary simple primitive affective feeling corresponding to a neurophysiological state that is continuously retrievable by the conscious mind [13]. A commonly used conceptualization is a three-factor structure categorizing core affect into valence, energetic arousal, and calmness [14].

In recent years, an increasing number of studies have examined the associations between daily physical behaviors and affective states, but the findings have been inconsistent in some cases [15]. For example, several observational studies have revealed that individual momentary affective states influence subsequent PB to the extent that higher ratings of the affective dimension valence led to a subsequent increase in daily activities [16–18]. However, some studies did not find an association between valence and subsequent increases in PA [19, 20]. It was further observed that an increase in valence was associated with a reduction in SB [21]. In contrast, one study found no alterations in subsequent SB [20]. Concerning energetic arousal, it was shown that higher levels led to increased PA [16–18, 20]. Consistent with these findings, studies found that energetic arousal predicted lower amounts of SB [20, 21]. Furthermore, a higher level of calmness led to a subsequent reduction in time spent physically active [16–18], whereas preceding higher levels of calmness predicted an increase in PA levels in one study [19]. An increase in calmness was associated with higher SB after the e-diary assessment [21]. Dunton speculated that the ambiguity might be caused by context-specific effects [22] and that future studies should consider those contextual factors more closely, suggesting that PB influences should be considered in multi-layered frameworks. The difficulty in promoting a physically active lifestyle lies in determining the drivers of SB, which could be used to develop effective behavior intervention strategies.

While PB has been known to assume a significant role in health behaviors, researchers have attempted to identify potential determinants e.g., other environmental factors that might influence physical behavior choices. In this context, environmental factors, such as weather conditions, are increasingly being addressed in a very topical way; for example, weather exposures that affect people's daily activities and indoor versus outdoor locations, such as increased temperature might be related to changes in PB. Future projections indicate an eight to 50-fold increase in the number of days that will be unsafe for moderate physical activity by 2070 [23]. Based on data from over 1.9 million people, Obradovich and Fowler (2017) have shown that both cold and acutely hot temperatures, as well as precipitation days, reduce PA [24]. Thus, weather conditions can impact everyday PB and in line with global climate change, there is a need to quantify how weather conditions influence decisions and the capacity to perform PB in daily life [25, 26].

Weather conditions might not only influence individuals' immediate decisions regarding PA but also might act as a potential moderator in the overall affective states and PB relationship. Several contextual factors such as natural, built, or social environments that may influence the time-dynamic, within-subject affect–behavior relationship have been investigated [27–29]. Self-perceived weather, such as too rainy, was shown to have a negative effect on affect, or perceived too cold weather reduced positive affect [30]. Individual affective responses to weather conditions were recently shown to be linked to mental well-being [25]. In general, contextual environmental factors have the potential to generate an additive effect on the association between affective states and PB. For example, even if people are in a positive mood and ready for a walk, as expected in previous research, the current rainy weather conditions may hinder them from going outside. Otherwise, people might be not energetic enough for being active, but the bright weather triggers them to go for a walk.

So far, theoretical and model-like explanations are based on affective and cognitive approaches [31–33]. In particular, dual-process models assume that behavior, in general, is regulated by two different mental processes: first by an implicit, automatic process that requires minimal cognitive resources (Type-1) and second by a slower and reflective, conscious process (Type-2) [32]. Regarding PB, automatically activated momentary affect is assumed to be sufficient to influence PA at a preconscious level [34, 35], independent of motivational attitudes [36]. In other words, such implicit Type-1 (affective) processes influence whether an active lifestyle/behavior is initiated or maintained [37]. To determine those Type-1 drivers of PA, investigating microtemporal within-subject processes is necessary [22].

Since affective states are dynamic in nature [38], it is essential to capture multiple affective states within a person over time [39]. Therefore, in our study, we used the method of ambulatory assessment (AA) [40], which is defined as the use of field methods to assess the ongoing behavior, physiology, experience, and environmental aspects of people in naturalistic or unconstrained settings [41]. The key advantages of AA are as follows: a) real-time assessments to overcome retrospective biases [42]; b) real-life assessments to increase ecological validity (reflect real-life conditions); c) repeated assessments reveal fluctuations in the processes of interest [43], thereby providing a within-subject perspective; d) the inclusion of time-varying covariates, such as contextual weather conditions, environment, and social context; and e) adding sophisticated interactive assessment such as activity or sedentary triggered e-diaries [44–46].

We are not aware of any study that integrated objective weather conditions combined with device-based assessment of PB and momentary affective states in real-time. Our study tries to illustrate how individual affective states and environmental factors (i.e., weather conditions) interact as determinants of PB. Therefore, we hypothesized that higher valence, higher energetic arousal, and lower calmness would lead to higher PA (hypothesis Ia) and less time spent in SB (hypothesis Ib). Second, we hypothesized that higher temperatures would increase PA and decrease SB (hypothesis IIa), whereas higher precipitation would decrease PA and increase SB (hypothesis IIb). Third, based on previous findings [30, 47–50], we investigated the additive effect of weather on the association between affective states and PB (exploratory interaction analyses III), namely whether contextual weather conditions (i.e., temperature and precipitation) have an interaction effect on the relationship between affective states and subsequent PB.

## Methods

### Participants

We recruited a convenience sample of university students and employees from September 2019 to March 2020 in Germany. Participants were included if they were able to perform daily activities without physical restrictions, had no currently present injuries, and reported no current mental disorders. In total, we recruited 111 participants. We excluded participants i) if their e-diary compliance was less than 30%, or due to technical issues (*N* = 4); ii) if they wore the accelerometers < / = 10 h and if the accelerometer was worn for fewer than three days (*N* = 12); iii) if their zip code did not match a particular weather station, that is, the residential distance had to be smaller than or equal to the distance of the weather station Rheinstetten, Germany to the furthest district of Karlsruhe (Grötzingen: 14.5 km) (*N* = 16); iv) if participants spent single days outside the weather station area (*N* = 33 days). The final sample consisted of 79 participants (60.8% females) between 17 and 49 years (22.7 ± 4.3 years) (for details, see Table 1). Written and oral information regarding the study procedures was presented to all eligible participants before written informed consent was obtained. All participants were free to withdraw from the study at any time. The ethics review board of the Karlsruhe Institute of Technology (KIT) approved this study.

**Table 1:** Participant and study characteristics (*N* = 79)

Variable	N; Mean ± SD^a^	Minimum	Maximum
Female [%]	48, 60.8%	–-	–-
Age [yrs.]	22.70 ± 4.26	17	49
BMI [kg/m^2^]	22.07 ± 2.00	17.21	26.85
Answered e-diary assessments [per day]^b^	4.76 ± 0.95	2.75	6.25
Answered e-diary assessments [%/5 days]^b^	65.97 ± 16.65	31.43	96.77
Calmness [0–100]^b^	67.51 ± 13.03	28.38	94.81
Valence [0–100]^b^	70.37 ± 12.26	39.14	95.96
Energetic Arousal [0–100]^b^	57.44 ± 11.91	22.46	85.83
Temperature [°C/day]^c^	7.84 ± 4.72	-1.82	18.83
Precipitation [mm/day]^d^	118.33 ± 202.60	0	1038
Wear time accelerometer [h/day]^c^	23.26 ± 1.53	13.70	24
Sedentary time [h/day] ^e^	9.60 ± 2.14	4.19	14.99
Movement acceleration [mg/day]^e^	45.65 ± 12.79	21.79	91.06

^a^standard deviation

^b^ assessed via e-diary, aggregated within participants

^c^ average per day across study period

^d^ sum per day across study period

^e^ aggregated within participants and days

### Study procedures

The assessment took place over five consecutive days from Wednesday to Sunday to cover weekdays and weekends. The participants received individualized training to become familiarized with the accelerometers (move 4) and the loaned smartphone (Nokia 6, Nokia Corporation, Espoo, Finland, Android phones) [51]. The participants wore the accelerometers at three different positions (i.e., wrist, hip, and thigh) for 24 h per day and answered e-diary questions between 8.00 am and 9.30 pm on weekdays and between 9.30 am and 10 pm on weekends.

### Study design

We used a mixed sampling strategy, combining activity-triggered assessments, sedentary-triggered assessments, and randomly triggered assessments. Technically, the thigh sensor analyzed and transmitted data on body position and movement acceleration to the smartphone in real-time via Bluetooth Low Energy.

Combined with random prompts, we used a sedentary-trigger algorithm that prompted the participants when they had spent ≥ 30 consecutive minutes in a sitting/lying body position (for details, see [46]), as well as an activity-triggered algorithm that prompted the participants if they spent ≥ 10 consecutive minutes of ≥ 220 mg (for details, see [45]). To reduce participant burden, time-out phases of 50 min were programmed, i.e., after an e-diary trigger, the participants would not receive another trigger in the following 50 min. If the prompt appeared at an inconvenient time, the participants had the option to postpone it by 5, 10, or 15 min. The three trigger conditions (sedentary, active, and random) were prompted until two of each were answered a day. This resulted in a maximum of six answered e-diary entries per day. The mixed sampling strategies and the e-diary assessments were implemented via movisensXS, version 0.7.47574 (movisens GmbH, Karlsruhe, Baden-Wuerttemberg, Germany).

### E-diary assessments

To assess momentary affect, we used the 6-item e-diary scale of Wilhelm and Schoebi, which is the first scale designed and evaluated solely for e-diary research [14]. It is constructed on a three-dimensional concept of affect, including valence, energetic arousal, and calmness. The six bipolar item scale is divided into these three respective affect dimensions: valence (item 5: unwell to well; item 2: discontent to content), energetic arousal (item 4: without energy to full energy; item 1: tired to awake) and calmness (item 6: tense to relaxed; item 3: agitated to calm). The bipolar items were displayed to the participants in a mixed order and with reversed polarity. The participants were able to express the varying degrees of their current affective state using a visual analog scale (0–100). The scale showed satisfying psychometric properties, both in the original publication [14] and in our dataset, with reliability coefficients on the within-person level ranging between 0.74 and 0.81 in our sample.

### Physical behavior measures

The Move 4 accelerometer is a validated device for measuring PB [52, 53]. The sensor measures triaxial acceleration with a range of ± 16 g, a sampling frequency of 64 Hz, and a resolution of 12 bits. The raw data from the sensor were saved to its internal memory card and bandpass filtered (0.25 to 11 Hz) to eliminate artifacts. PA and SB were calculated from the accelerometer's raw files in 1-min epochs using the software DataAnalyzer (version 1.13.7; movisens GmbH). PA was operationalized as the aggregated triaxial mean movement acceleration intensity in the gravitational constant g (9.81 m/s2) in 10 min segments after each e-diary entry, according to published procedures [16, 18]. The outcome variable mg indicates the intensity of PB (e.g., jogging is about 1103 mg/min, walking is 367 mg/min and sitting 7 mg/min [53]). SB was parameterized as the number of aggregated sedentary minutes within the 30 min time frame after each e-diary entry [21].

### Meteorological measures

Meteorological data were retrieved from a publicly available dataset from the Climate Data Center of the German Weather Service (Deutscher Wetterdienst; DWD) [54, 55]. Specifically, we obtained hourly values for temperature [°C] and precipitation [mm] from a weather station in Rheinstetten [56], which is located approximately seven kilometers from the Karlsruhe Institute of Technology (station id: 4177; longitude: 48.5847; latitude: 8.1952, height: 116 m). We aligned temperature values to 60 min intervals before each e-diary assessment.

### Data analysis

PA and SB data were merged with the ratings of the e-diary entries (using the software DataMerger, version 1.8.0, movisens GmbH) and the hourly weather parameters. The outcome parameter movement acceleration was log-transformed using a natural logarithm since the distribution was right-skewed. The skew statistic was 4.07, and after applying log-transformation to the PA data, skewness was reduced to 0.22.

Multilevel models were conducted to examine momentary within-person effects of affective states on subsequent PB outcomes [57]. In the calculated models, repeated measurements (on level-1) within participants (on level-2) were nested. First, we estimated the intraclass correlation coefficient (ICC), whereby the logarithmized values of movement acceleration [mg] and SB [min] served as dependent variables to indicate the amount of variance on the within- vs. between-person level by calculating unconditional (null-) models. Second, we added the time-variant and time-invariant predictors time [hour], time-squared [hours^2^], age [years], sex [female vs. male], calmness [0–100], energetic arousal [0–100], valence [0–100] and BMI [m/kg^2^] to our main model (see equation for hypothesis Ia below).

Model 1 (hypothesis Ia):

$$Y{\left(movement\;acceleration\right)}_{ij}=\gamma_{00}+\gamma_{01}\ast{age}_j+\gamma_{02}\ast{BMI}_j+\gamma_{03}\ast{sex}_j+\gamma_{10}\ast{valence}_{ij}+\gamma_{20}\ast{energetic\;arousal}_{ij}+\gamma_{30}\ast{calmness}_{ij}+\gamma_{40}\ast{time\;of\;day}_{ij}+\gamma_{50}\ast{time\;of\;day}_{ij}^2+r_{ij}$$

On level 1, within-subject effects were estimated through participants' e-diary entries (subscript _j_) at each measurement time point (subscript _i_). Y_ij_ reflects the level of movement acceleration in person *j* at the time *i*. At level 1, beta coefficients represent the intercept (γ_00_) and the effects of time, time-squared, within-subject valence, energetic arousal, and calmness (γ_10_- γ_50_). At level 2, beta coefficients represent time-invariant covariates (γ_01_- γ_03_). r_ij_ represents the residuals at level 1. Additionally, a multilevel random-intercept model was conducted to test whether the type of trigger (i.e., random vs. PB-triggered) might influence our main findings from hypotheses (Ia-IIb). Third, we added the weather parameters temperature [°C] and precipitation [mm] to our main model as time-variant predictors. Fourth, we conducted exploratory interaction analyses for PA and SB combining all possible interactions between affective states and weather parameters (i.e., resulting in twelve different interactions) (see equation in Additional file 1). We centered the affective states and weather predictors on a personal level. Significant random effects were included in our models. We specified our models by using restricted maximum likelihood (REML) as the model estimator and unstructured as covariance structure. To compare each predictor's effects, we calculated standardized beta coefficients (stand. β) following established procedures [54]. To compare the model fit, we used the -2ΔLL likelihood ratio test. To calculate the proportion of explained total outcome variance, we used the predicted outcome's squared correlation (R2) by using the fixed effects and actual values. Following established simulation studies towards multilevel power analysis [58], our data are suited for the detection of small within-subject effects and medium to large between-subject effects. To draw practical conclusions concerning the impact of alterations in contextual dimensions on PA, the percentage change rates were calculated using the following equation (see [16]). A 1-point increase in temperature (C°) would lead to a percentage change in PA by

$$\delta =\left(\left({e}^{\beta \left(temperature\right)\times 1}\right)-1\right)\times 100$$

For all analyses, the α-level was set to 0.05. All data analyses were conducted with SPSS software (IBM), version 26.0.0.0. We followed the strengthening the reporting of observational studies in epidemiology (STROBE) reporting guidelines (see Additional file 2) in reporting analyses and results.

## Results

### Descriptive statistics

In total, 2537 e-diary prompts were sent. On average, the 79 participants received 32 (range = 14–47; SD = 6.61) prompts during the study week, of which the participants responded to an average of 20.8 prompts over the five days (see Table 1). This resulted in an overall compliance of 66% (SD = 16.65). The average score reported by the participants for valence was 70.37 (SD = 12.26), for energetic arousal was 57.44 (SD = 11.91), and for calmness was 67.51 (SD = 13.03) on a scale of 0–100. The intraclass correlation coefficient (ICC) showed that 90.58% of SB (30 min timeframe) and 91.79% of PA (10 min timeframe) were due to within-subject fluctuations within the sample. The accelerometers were worn for an average of 23.26 (SD = 1.53) hrs per day per participant. Furthermore, the mean PA across participants was 45.65 mg (range = 21.79–91.06; SD = 12.79), and the mean time spent in SB was 9.6 h (range = 4.19–14.99; SD = 2.14) per day. The daily mean temperature ranged from -1.82 to 18.83 °C, with an average of 7.84 °C (SD = 4.72). The daily sum of precipitation ranged from 0 to 1038 mm (mean 118.33; SD = 202.60; 1000 mm equals 1 l/m^2^). The 60 min timeframes of weather parameters prior to the e-diary prompts ranged from -3.42 to 25 °C (temperature) and from 0 to 3.93 mm (precipitation), respectively.

#### Association of affective states with physical behavior 

Confirming hypothesis Ia, higher levels of valence and energetic arousal predicted higher levels of PA (stand. β = 0.134, *p* < 0.001; stand. β = 0.156, *p* < 0.000, respectively). That is, momentary ratings of valence and energetic arousal were positively associated with the subsequent movement acceleration within the 10 min after e-diary assessments. Translated to practice, on average, higher momentary ratings of valence (e.g., 80) compared to lower ratings (e.g., 20) were associated with subsequent higher levels of PA (12.23 mg). On average, higher ratings of energetic arousal (e.g., 80) compared to lower ratings (e.g., 20) were associated with subsequent higher amounts of PA of about 13.26 mg. As hypothesized, an increase in calmness negatively predicted movement acceleration (stand. β = -0.130, *p* < 0.001). This means, when participants felt more relaxed and calm (e.g., 80) compared to lower calmness ratings (e.g., 20), on average, their subsequent PA was 14.47 mg lower in the 10 min after each e-diary prompt. None of the other predictors (age, sex, BMI, time, time-squared) showed any significant influence on PB. We found no significant random effects for any predictors. A robust analysis showed that the main findings of our models (1-3b) were independent of the type of trigger (random vs. PB-triggered). In particular, adding the type of trigger as a covariate to the reported models did not change any significant values of our main findings. The results are presented in Table 2.

**Table 2:** Multilevel model analyses to predict physical behavior: fixed and random effects

		***PA models***				***SB models***		
		**Model 1**	**Model 2**	**Model 3**	**Model 3**	**Model 1b**	**Model 2b**	**Model 3b**
		b /stand. b (SE)	b /stand. b (SE)	b /stand. b (SE)	b /stand. b (SE)	b /stand. b (SE)	b /stand. b (SE)	b /stand. b (SE)
**Fixed effects**	Intercept, ${\beta }_{00}$	3.393** (.570)	3.499** (.571)	3.519** (.575)	3.514** (.571)	22.167** (5.810)	21.450** (5.773)	21.438** (5.870)
	Sex^a^, ${\beta }_{03}$	.076/.037 (.090)	.076/.036 (.090)	.073/.036 (.091)	.077/.038 (.090)	-.199/-.010 (.915)	-.202/-.009 (.908)	-.233/-.011 (.923)
	Age, ${\beta }_{01}$	-.009/-.025 (.010)	-.009/-.024 (.010)	-.009/-.026 (.010)	-.009/-.026 (.010)	-.175/-.048 (.099)	-.173/-.049 (.098)	-.173/-.047 (.100)
	BMI, ${\beta }_{02}$	-.017/-.033 (.022)	-.017/-.033 (.022)	-.016/-.032 (.022)	-.017/-.033 (.022)	-.029/-.006 (.226)	-.028/-.005 (.224)	-.029/-.006 (.228)
	Calmness, ${\beta }_{30}$	-.006**/-.130 (.002)	-.006**/-.128 (.002)	-.006**/-.126 (.002)	-.006**/-.127 (.002)	.054*/.109 (.018)	.051**/.109 (.018)	.054**/.108 (.018)
	Valence, ${\beta }_{10}$	.007**/.134 (.002)	.007**/.137 (.002)	.007**/.140 (.002)	.007**/.141 (.002)	-.026/-.049 (.021)	-.025/-.051 (.021)	-.028/-.054 (.021)
	Energetic Arousal, ${\beta }_{20}$	.007**/.156 (.001)	.007**/.157 (.001)	.007**/.156 (.001)	.007**/.161 (.001)	-.017/-.038 (.014)	-.019/-.039 (.014)	-.019/-.041 (.014)
	Temperature, ${\beta }_{60}$	–-	.025/.139* (.010)	.026**/.138 (.009)	.025**/.136 (.009)	–-	-.146/-.084 (.086)	-.150/-.081 (.086)
	Precipitation, ${\beta }_{70}$	–-	.007/.003 (.081)	.008/.003 (.081)	.005/.002 (.081)	–-	-.366/-.014 (.768)	-.365/-.014 (.767)
	Calmness*temperature, ${\beta }_{80}$	–-	–-	.001*/.072 (.001)	–-	–-	–-	–-
	Valence*temperature, ${\beta }_{80}$	–-	–-	–-	.001*/.075 (.001)	–-	–-	-.011*/-.066 (.005)
	Time of day, ${\beta }_{40}$	.069**/.265 (.025)	.032/.120 (.028)	.027/.102 (.028)	.030/.114 (.028)	.034/.013 (.244)	.267/.101 (.277)	.276/.106 (.273)
	Time of day squared, ${\beta }_{50}$	-.005**/-.260 (.002)	-.002/-.124 (.002)	-.002/-.108 (.002)	-.002/-.116 (.002)	.009/.053 (.017)	-.006/-.029 (.019)	-.006/-.036 (.019)
**Random effects**	Intercept, ${u}_{0}$	.087** (.022)	.087** (.022)	.089** (.023)	.087** (.022)	9.663** (2.428)	9.475** (2.409)	.089** (.023)
	Residual, $\mathrm{r}$	.885** (.033)	.870** (.033)	.878** (.032)	.878** (.032)	90.753** (3.223)	89.106** (3.229)	.878** (.032)

*Note*^*1*^*:* Unstandardized / standardized estimates and standard errors

^a^compared to males

^*^ *P* < .05

^**^ *P* < .01

The affective states valence and energetic arousal did not significantly predict sedentary time in the consecutive 30 min to the e-diary prompt (stand. β = -0.050, *p* = 0.226; stand. β = -0.038, *p* = 0.209, respectively); thus, hypothesis Ib regarding valence and energetic arousal was not verified. As it was assumed, higher levels of calmness significantly predicted more time spend sedentary in 30 min following the e-diary prompt (stand. β = 0.109, *p* = 0.003), thus verifying hypothesis Ib regarding calmness. In practice, a 1-point increase in calmness (scale: 0–100) led to an increase in SB of 0.05 min in the 30 min timeframe following the e-diary prompt.

#### Association of contextual weather conditions on physical behavior 

In our second model, we tested whether contextual weather conditions predicted within-subject variations in PA intensity or time spent in SB. According to hypothesis IIa, we found a significant positive association between temperature and PA intensity. In detail, higher degrees of temperature positively influenced PA intensity (stand. β = 0.139, *p* = 0.005). There was no significant association between the amount of hourly precipitation on the subsequent PA intensity (stand. β = 0.003, *p* = 0.967) (hypothesis IIb). In addition, we found no significant within-subject associations between the predictors temperature (stand. β = -0.084, *p* = 0.156) and precipitation (stand. β = -0.014, *p* = 0.633) on time spent in SB (hypotheses IIa and IIb).

#### Exploratory interaction analyses 

**Fig. 1:**
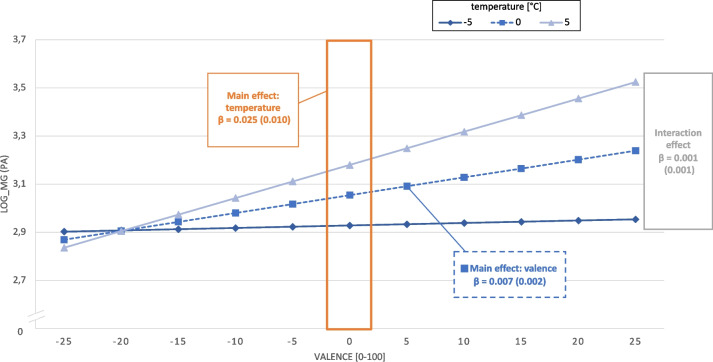
Beta coefficients of the ML model demonstrating the main effect of valence, the main effect of temperature and the interaction of valence*temperature on physical activity. Y-axis: log-transformed mg values; X-axis person-centered valence

Three out of twelve exploratory multilevel models (3 affective states * 2 contextual weather conditions predicting 2 physical behaviors) revealed a significant interaction of affective states and contextual weather conditions on PB. In particular, temperature positively moderated the association between valence and PA (stand. β = 0.075, *p* = 0.036). This is depicted in Fig. 1, showing all three effects, namely, the two main effects as well as the interaction. First, the main effect of temperature on PA is depicted in the orange area. A 5 °C temperature above the personal average would increase subsequent activity behavior by approximately 12.5% (light blue triangle), whereas a 5 °C temperature below the personal average would predict lower PA (dark blue rhombus). Second, the main effect of valence on PA is shown by the dashed blue line. If valence is rated higher by five points, the subsequent activity behavior in the 10 min after the e-diary prompt was increased by approximately 3.5%. Third, the interaction effect, which is the effect of valence on PA moderated by temperature, is depicted by the three different slopes (light blue triangle, dashed blue square, dark blue rhombus). A 5 °C temperature above the personal average was linked to a steeper association between valence and PA (light blue triangle) compared to the average context (dashed blue line with squares), whereas in the 5 °C temperature below the personal average condition, the slope is much lower (dark blue rhombus line). In other words, the temperature enhances the effect of valence on PA.

**Fig. 2:**
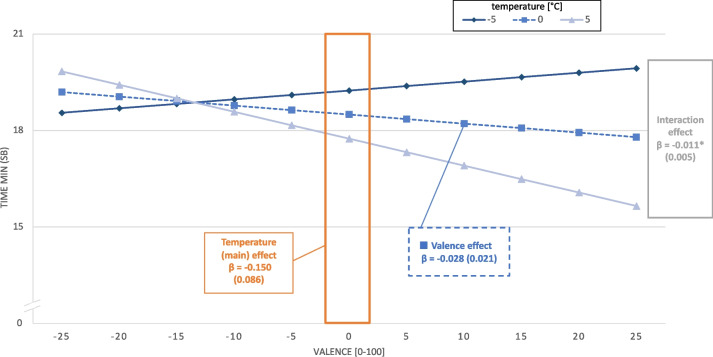
Beta coefficients of the ML model demonstrating the main effect of valence, the main effect of temperature and the interaction of valence*temperature on sedentary behavior. Y-axis: amount of time (min) spent in sedentary behavior; X-axis: person-centered valence

The second interaction model showed that temperature negatively moderated the association between valence and SB (stand. β = -0.066, *p* = 0.043), as demonstrated in Fig. 2. The main effect of temperature on SB is depicted in the orange area, showing that 5 °C above the personal average was associated with a decrease in subsequent SB, whereas 5 °C below the personal average showed the opposite effect. The main effect of valence on SB was negative and shown by the dashed blue line. Increasing valence was related to less SB (non-significant). The interaction, which was the effect of valence on SB moderated by temperature, is depicted by the three different slopes. A 5 °C temperature above the personal average is linked to a steeper negative association between valence on SB (light blue triangle), whereas in the 5 °C temperature below the personal average condition, the slope is positive (dark blue rhombus). In other words, the effect of positive valence reducing SB was enhanced at higher temperatures.

**Fig. 3:**
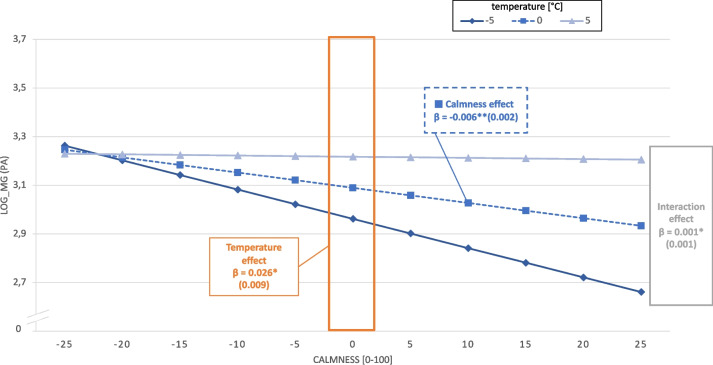
Beta coefficients of the ML model demonstrating the main effect of calmness, the main effect of temperature and the interaction of calmness*temperature on physical activity. Y-axis: log-transformed mg values; X-axis: person-centered calmness

Furthermore, the third significant interaction model showed that the effect of calmness on PA was moderated by temperature (stand. β = 0.072, *p* = 0.019), which is depicted in Fig. 3. The main effect of temperature on PA is depicted in the orange area. A temperature 5 °C above the personal average increased subsequent PA by approximately 13% (light blue triangle), whereas a temperature 5 °C below the personal average predicted lower PA (dark blue rhombus). The negative association between calmness and PA is shown by the dashed blue line, with a 5-point increase in calmness resulting in 3% less PA. The interaction, which is the effect of calmness on PA moderated by temperature, is depicted by the three different slopes. A 5 °C temperature above the personal average was linked to a less pronounced association between calmness and PA (light blue triangle), whereas in the 5 °C temperature below the personal average condition, the slope is more negative (dark blue rhombus line). Oversimplified, the negative effect of calmness on PA dissolves with higher temperatures.

## Discussion

To better understand how the interplay between affective states and contextual weather conditions drives our everyday life PB, we investigated momentary affective states (i.e., valence, energetic arousal, and calmness), contextual factors (i.e., precipitation and temperature), and PB in a sample of healthy adults. There was some evidence that the association between affective states and PA was moderated by temperature. In addition, we found associations between momentary contextual weather conditions and PA, and we confirmed earlier findings associating affective states with PB. Our exploratory interaction analyses have shown that contextual weather conditions slightly moderated the association between within-subject affective states and PB in three models. In detail, a higher temperature tentatively a) enhanced the positive effect of valence on PA, b) enhanced the effect of valence on reducing SB, and c) dissolves the negative effect of calmness on PA.

We confirmed that higher ratings of momentary energetic arousal and valence led to subsequent increases in PA intensity, whereas momentary calmness led to reductions in subsequent PA intensity (hypothesis Ia). These findings are in line with most previous studies that used similar methods [15]. In particular, several studies examined the short-term association with positive affect and the duration of accumulated subsequent PA levels (e.g., 10–30 min) provide evidence for positive affect predicting increased PA [59–61]. The same associations between affective states (i.e., valence, energetic arousal, calmness) and non-exercise activity were revealed by Reichert and colleagues [18], Kanning and Schoebi [17] in adults as well as by Koch and colleagues [16] in adolescents. These studies also applied short time frames for their analyses (i.e., 10–45 min). However, Kim and colleagues [20] used extended time intervals (PB up to 120 min after ratings of affect) and rarely found an association between affective states and PB, whereas Stavrakakis and colleagues [62] utilized 360 min revealing no associations in the affective dimension positive affect and another study found no associations of positive affect influencing subsequent PA after 1440 min [63]. Future studies might give greater consideration to the temporal resolution of associations between PB and affective states.

In contrast to our hypothesis Ib, momentary calmness but not valence and energetic arousal significantly predicted upcoming SB. In detail, higher momentary valence was not associated with subsequent device-measured SB, which was consistent with Kim and colleagues [20] in a sample of working adults but not consistent with several other studies showing that elevated valence was associated with decreased sedentariness [21, 64, 65]. We also revealed no association of energetic arousal with subsequent SB. This finding was supported by some other studies [65, 66], but contrary to Giurgiu and colleagues [21] and Kim and colleagues [20] that showed a relation between higher activation and reduced sedentary time. In line with our expectations, higher momentary calmness was associated with subsequently lower amounts of SB, thus confirming hypothesis Ib. Another study investigated the associations of calmness with SB and thus supported our findings [21].

The diverse findings might be due to a high degree of heterogeneity in the methodological assessments of PB and affect (e.g., accelerometer location, parameterization of SB, sample design, applied questionnaire, and item selection). We closely followed the methodological approach of previous studies [16, 18, 21] to enable comparability. Replication of methods and study designs is highly needed for establishing the reproducibility and generalizability of the findings and for future meta-analyses to integrate findings. Moreover, future research endeavors might be interested in researching a reciprocal nature between affective states and PB and their interactions with weather conditions, e.g., by applying dynamic structural equation modeling and through exploration of potentially more time-enduring (‘trait-like’) effects. Further analyses revealed that there was an association between temperature with PA, but not with SB (hypothesis IIa). In detail, temperature was independently positively associated with subsequent PA. This finding was supported by three studies that also objectively assessed temperature. In a sample of older adults, lower temperatures resulted in up to 10% less PA [67], and higher temperatures increased walking and cycling among adults [68, 69]. Studies with self-defined perceptions of weather conditions also support our findings [30, 63]. In contrast to our hypothesis, the amount of precipitation was not associated with PB (hypothesis IIb). Our results were in line with a study that examined daily precipitation and its influence on PA in over 200 adults and found no significant association [70]. Furthermore, in a sample of elderly individuals, Colom and colleagues [71] found no association between precipitation measured at the daily level and PA - either objectively measured via accelerometer or self-reported. However, in a study with COPD patients over 12 months, it was shown that the hours of rain per day were negatively associated with the number of steps taken [72].

In contrast to our hypothesis (IIb), we found no association between contextual weather conditions and SB, which is, at least partially, in contrast to the literature. In detail, Yildirim and colleagues [73] found a positive significant relationship between precipitation and SB in a sample of *N* = 722 children. Sartini and colleagues [74] found a positive relation between SB and temperature in 1361 elderly individuals. One study revealed that SB was reduced when weather conditions change from rainy to sunny days [75], whereas another found an association between SB and temperature only on weekends [76]. However, in all those studies, meteorological data were analyzed on a daily basis, rather than an hourly basis as in our study.

How can our null findings be explained, given that our methods might be superior (hourly meteorological data; multisensor system to detect sedentariness). One possible explanation might be restricted variance. Our sample consisted of working adults who may exhibit a maximum of SB due to their work life. This might be less extreme in other studies with children and adolescents [48] and elderly individuals [74]. In addition, precipitation was quite low in our sample. However, having a higher timely resolution might mitigate this issue, thereby increasing variance over time. Additionally, it is theoretically possible that momentary weather conditions may influence a person's activity behavior more than average weather conditions throughout the day. Another explanation for the positive findings of the other studies might be their approach to detect SB. Using a wrist sensor might conflate true SB with PA and bias standing and sitting phases [52], thereby revealing associations between both constructs.

To the best of our knowledge, this is the first study that investigated moderation effects based on device-based data, whereas previous studies investigating the subjective appraisal of self-perceived weather conditions [30] in relation to PB on a daily level [63] revealed inconclusive results, which prevents us from discussing our findings against the background of the literature. However, our results may provide potential implications for future real-life interventions if replicated and verified to be of causal nature. It should be noted that the effects we found were small and thus should be interpreted with caution for further recommended actions. One may be tempted to speculate that intervention strategies on affective valence to increase PA and to decrease sedentary time should be especially applied at times and in settings with an above-average air temperature. According to our results, influencing affective valence in this context (e.g., via emotion regulation strategies such as mindfulness training) might be especially promising to counteract physical inactivity. Moreover, we are tempted to speculate that interventions on feelings of stress and calmness aiming to alter PA seem to be more promising when air temperature is below average since our findings showed calmness to scarcely be associated with PA in contexts of high air temperature. As a next step beyond replication and causality testing, differences between persons in these complex interactions should be researched. In summary, tailoring mobile interventions based not only on psychological determinants of PB but also on contextual influences such as weather conditions and especially on the interaction of psychological determinants with contextual influences may offer promising avenues for just-in-time adaptive interventions (JITAI) [77] to reduce physical inactivity. For example, future research endeavors might be interested in developing a “physical activity prediction” application or trigger based on the current mood and the upcoming weather forecast. With regard to changes in climatic conditions, awareness has to be raised that environmental factors might influence the promotion of PA. So far, the influences of seasonal conditions and weather factors have received little attention in the promotion of PA [78]. In our study, we demonstrated that temperature alterations might have the potential to influence subsequent PA. If this were to be the case, for example, community sports settings and outdoor summer sports may be most affected by climatic changes [23, 26]. This could be a pertinent indication for the development of health policy strategies to counteract this progress, as weather phenomena may be a deterrent for people to engage in PA.

Some limitations must be considered. First, our sample was recruited from university staff, which may hinder generalization to the general population. Also, larger sample sizes might be superior in terms of generalizability and between-subject effects. Second, our observed associations between affective states, PB, and weather conditions included data from September to March during the European fall and winter seasons, thus, limiting a generalization to other geographic areas with different weather conditions and PB. Furthermore, the within-subject variability in precipitation was quite limited in our sample, which might have increased the difficulty of revealing meaningful associations. Future studies should consider potential seasonal effects, prolonged periods of time covering different seasons, and locations with higher precipitation. Third, we assessed contextual weather conditions objectively with a high temporal resolution. It is assumed that contextual weather conditions are linked to PA in an inverted u-shaped pattern, indicating that PA within individuals decreases under extreme weather conditions (e.g., ≥ 30 C°) [79–82]. Sound empirical evidence for those assumptions would require long-term monitoring across all seasons, and probably, the subjective perception of weather conditions. Assessing the individual subjective feeling of weather conditions (e.g., too cold, too hot) in addition to objective weather data from local stations might be a favorable add-on in future studies. Another limitation inherent in our study pertains to the exclusion of participants and single days in which participants did not reside within the proximity of the weather station. Future studies could address this issue by incorporating GPS data to track participants' location. Fourth, chronology only represents one aspect of causality [83]. Chronology suggests causality though does not prove it, as hidden third variables could have similar time-related characteristics. Additional studies are needed to support a causal hypothesis; one approach could be to employ ecological momentary interventions (EMI) to experimentally induce effects of PB in everyday life [84], potentially taking into account weather effects.

## Conclusions

In this AA study, we investigated how within-subject affective states as well as weather conditions are associated with subsequent PB, using recurring real-time and real-life assessments of affective states combined with device-based measured PB in the everyday life of a community-based sample. We also explored the interaction of affective states, weather conditions, and PB. We found that both momentary valence and energetic arousal were positively related to subsequent PA whereas calmness led to reductions in PA within the subsequent timeframe. In particular, the more participants felt content, energetic, and less calm in everyday life, the more they were physically active. Momentary calmness significantly predicted upcoming SB – in practice, if participants felt calmer and more relaxed, they engaged in more sedentary time. We also found temperature positively related to subsequent PA. Participants tended to be more physically active when temperatures were higher within the German fall and winter season, characterized by rather mild temperatures (10 C°/4.2 C° [85]). Furthermore, our exploratory interaction analyses showed small effects of weather conditions moderating the association between within-subject affective states and PB. In detail, higher temperatures enhanced the positive effect of valence on PA and on reducing SB and also dissolves the negative effect of calmness on PA. Long-term studies including vulnerable groups and covering additional climate zones are needed to observe the associations between weather variables and PB. Future studies could identify at which environmental condition a decrease in PA occurs—for example, due to heat waves or air pollution [86]. Moreover, JITAIs could be assessed to encourage PA as they offer the possibility to incorporate momentary affect or contextual factors, such as weather conditions, in real-time and allow triggering individuals within their preferred conditions to promote PA. Such personalized real-time prompts should theoretically be superior and require an understanding of the influence of affect dimensions and weather conditions on an individual’s behavior. JITAIs can incorporate opportunities for PA that have obscured the effectiveness of interventions in promoting PA. This may contribute to people adopting a physically active lifestyle, which can contribute to overall improved health outcomes.

### Supplementary Information

**Additional file 1.**

**Additional file 2.** STROBE Statement.

## Data Availability

The datasets during and/or analyzed during the current study available from the corresponding author on reasonable request.

## Abbreviations

AA: Ambulatory assessment
BMI: Body mass index
JITAI: Just-in-time adaptive intervention
Mg: Movement acceleration
PA: Physical activity
PB: Physical behavior
SB: Sedentary behavior
