# Supplementary material for: Momentary within-subject associations of affective states and physical behavior are moderated by weather conditions in real life: an ambulatory assessment study
Source: Int J Behav Nutr Phys Act. 2023 Sep 30;20:117. doi: 10.1186/s12966-023-01507-0 (PMC10541720; doi:10.1186/s12966-023-01507-0)
Supplement: Supplementary file 1 — Additional file 1. [file 12966_2023_1507_MOESM1_ESM.docx]

Additional file 1.

Model 1:

$${\text{Y}\left( \text{movement acceleration} \right)}_{\text{ij}}\text{=}\text{γ}_{\text{00}}\text{+ }\text{γ}_{\text{01}}\text{*}\text{age}_{\text{j}}\text{+}\text{γ}_{\text{02}}\text{*}\text{BMI}_{\text{j}}\text{+}\text{γ}_{\text{03}}\text{*}\text{sex}_{\text{j}}\text{+}\text{+ γ}_{\text{10}}\text{*}{\text{valence}_{\text{ij}}\text{ }\text{+ γ}_{\text{20}}\text{*}\text{energetic arousal}_{\text{ij}}\text{+ }\text{γ}_{\text{30}}\text{*calmness}}_{\text{ij}}\text{γ}_{\text{40}}\text{*}\text{time of day}_{\text{ij }}\text{+ }\text{γ}_{\text{50}}\text{*}\text{time of day}_{\text{ij}}^{\text{2}}\text{ +}\text{r}_{\text{ij}}$$

$${\text{Y}\left( \text{sedentary time} \right)}_{\text{ij}}\text{=}\text{γ}_{\text{00}}\text{+ }\text{γ}_{\text{01}}\text{*}\text{age}_{\text{j}}\text{+}\text{γ}_{\text{02}}\text{*}\text{BMI}_{\text{j}}\text{+}\text{γ}_{\text{03}}\text{*}\text{sex}_{\text{j}}\text{+}\text{+ γ}_{\text{10}}\text{*}{\text{valence}_{\text{ij}}\text{ }\text{+ γ}_{\text{20}}\text{*}\text{energetic arousal}_{\text{ij}}\text{+ }\text{γ}_{\text{30}}\text{*calmness}}_{\text{ij}}\text{γ}_{\text{40}}\text{*}\text{time of day}_{\text{ij }}\text{+ }\text{γ}_{\text{50}}\text{*}\text{time of day}_{\text{ij}}^{\text{2}}\text{ +}\text{r}_{\text{ij}}$$

Model 2:

$${\text{Y}\left( \text{movement acceleration} \right)}_{\text{ij}}\text{=}\text{γ}_{\text{00}}\text{+ }\text{γ}_{\text{01}}\text{*}\text{age}_{\text{j}}\text{+}\text{γ}_{\text{02}}\text{*}\text{BMI}_{\text{j}}\text{+}\text{γ}_{\text{03}}\text{*}\text{sex}_{\text{j}}\text{+ γ}_{\text{10}}\text{*}{\text{valence}_{\text{ij}}\text{ }\text{+ γ}_{\text{20}}\text{*}\text{energetic arousal}_{\text{ij}}\text{+ }\text{γ}_{\text{30}}\text{*calmness}}_{\text{ij}}\text{+}\text{γ}_{\text{40}}\text{*}\text{time of day}_{\text{ij }}\text{+ }\text{γ}_{\text{50}}\text{*}\text{time of day}_{\text{ij}}^{\text{2}}\text{ + }\text{γ}_{\text{60}}\text{*}\text{temperature}_{\text{ij}}\text{+ }\text{γ}_{\text{70}}\text{*}\text{precipitation}_{\text{ij}}\text{+}\text{r}_{\text{ij}}$$

$${\text{Y}\left( \text{sedentary time} \right)}_{\text{ij}}\text{=}\text{γ}_{\text{00}}\text{+ }\text{γ}_{\text{01}}\text{*}\text{age}_{\text{j}}\text{+}\text{γ}_{\text{02}}\text{*}\text{BMI}_{\text{j}}\text{+}\text{γ}_{\text{03}}\text{*}\text{sex}_{\text{j}}\text{+ γ}_{\text{10}}\text{*}{\text{valence}_{\text{ij}}\text{ }\text{+ γ}_{\text{20}}\text{*}\text{energetic arousal}_{\text{ij}}\text{+ }\text{γ}_{\text{30}}\text{*calmness}}_{\text{ij}}\text{+}\text{γ}_{\text{40}}\text{*}\text{time of day}_{\text{ij }}\text{+ }\text{γ}_{\text{50}}\text{*}\text{time of day}_{\text{ij}}^{\text{2}}\text{ + }\text{γ}_{\text{60}}\text{*}\text{temperature}_{\text{ij}}\text{+ }\text{γ}_{\text{70}}\text{*}\text{precipitation}_{\text{ij}}\text{+}\text{r}_{\text{ij}}$$

Model 3:

$${\text{Y}\left( \text{movement acceleration} \right)}_{\text{ij}}\text{=}\text{γ}_{\text{00}}\text{+ }\text{γ}_{\text{01}}\text{*}\text{age}_{\text{j}}\text{+}\text{γ}_{\text{02}}\text{*}\text{BMI}_{\text{j}}\text{+}\text{γ}_{\text{03}}\text{*}\text{sex}_{\text{j}}\text{+ γ}_{\text{10}}\text{*}{\text{valence}_{\text{ij}}\text{ }\text{+ γ}_{\text{20}}\text{*}\text{energetic arousal}_{\text{ij}}\text{+ }\text{γ}_{\text{30}}\text{*calmness}}_{\text{ij}}\text{+}\text{γ}_{\text{40}}\text{*}\text{time of day}_{\text{ij }}\text{+ }\text{γ}_{\text{50}}\text{*}\text{time of day}_{\text{ij}}^{\text{2}}\text{ + }\text{γ}_{\text{60}}\text{*}\text{temperature}_{\text{ij}}\text{+ }\text{γ}_{\text{70}}\text{*}\text{precipitation}_{\text{ij}}\text{ +}\text{γ}_{\text{80}}\text{*}\text{valence}_{\text{ij }}\text{*}\text{temperature}_{\text{ij }}\text{+}\text{r}_{\text{ij}}$$

$${\text{Y}\left( \text{movement acceleration} \right)}_{\text{ij}}\text{=}\text{γ}_{\text{00}}\text{+ }\text{γ}_{\text{01}}\text{*}\text{age}_{\text{j}}\text{+}\text{γ}_{\text{02}}\text{*}\text{BMI}_{\text{j}}\text{+}\text{γ}_{\text{03}}\text{*}\text{sex}_{\text{j}}\text{+ γ}_{\text{10}}\text{*}{\text{valence}_{\text{ij}}\text{ }\text{+ γ}_{\text{20}}\text{*}\text{energetic arousal}_{\text{ij}}\text{+ }\text{γ}_{\text{30}}\text{*calmness}}_{\text{ij}}\text{+}\text{γ}_{\text{40}}\text{*}\text{time of day}_{\text{ij }}\text{+ }\text{γ}_{\text{50}}\text{*}\text{time of day}_{\text{ij}}^{\text{2}}\text{ + }\text{γ}_{\text{60}}\text{*}\text{temperature}_{\text{ij}}\text{+ }\text{γ}_{\text{70}}\text{*}\text{precipitation}_{\text{ij}}\text{ +}\text{γ}_{\text{80}}\text{*}\text{calmness}_{\text{ij }}\text{*}\text{temperature}_{\text{ij }}\text{+}\text{r}_{\text{ij}}$$

$${\text{Y}\left( \text{sedentary time} \right)}_{\text{ij}}\text{=}\text{γ}_{\text{00}}\text{+ }\text{γ}_{\text{01}}\text{*}\text{age}_{\text{j}}\text{+}\text{γ}_{\text{02}}\text{*}\text{BMI}_{\text{j}}\text{+}\text{γ}_{\text{03}}\text{*}\text{sex}_{\text{j}}\text{+ γ}_{\text{10}}\text{*}{\text{valence}_{\text{ij}}\text{ }\text{+ γ}_{\text{20}}\text{*}\text{energetic arousal}_{\text{ij}}\text{+ }\text{γ}_{\text{30}}\text{*calmness}}_{\text{ij}}\text{+}\text{γ}_{\text{40}}\text{*}\text{time of day}_{\text{ij }}\text{+ }\text{γ}_{\text{50}}\text{*}\text{time of day}_{\text{ij}}^{\text{2}}\text{ + }\text{γ}_{\text{60}}\text{*}\text{temperature}_{\text{ij}}\text{+ }\text{γ}_{\text{70}}\text{*}\text{precipitation}_{\text{ij}}\text{+}\text{γ}_{\text{80}}\text{*}\text{valence}_{\text{ij}}\text{*}\text{temperature}_{\text{ij}}\text{+}\text{r}_{\text{ij}}$$
