# Supplementary material for: Momentary within-subject associations of affective states and physical behavior are moderated by weather conditions in real life: an ambulatory assessment study
Source: Int J Behav Nutr Phys Act. 2023 Sep 30;20:117. doi: 10.1186/s12966-023-01507-0 (PMC10541720; doi:10.1186/s12966-023-01507-0)
Supplement: Supplementary file 2 — Additional file 2. STROBE Statement. [file 12966_2023_1507_MOESM2_ESM.docx]

STROBE Statement—Checklist of items that should be included in reports of observational studies

|  | Item No | Recommendation | Page No. |
| --- | --- | --- | --- |
| **Title and abstract** | 1 | (*a*) Indicate the study’s design with a commonly used term in the title or the abstract | p. 1; lines 1-3 |
|  |  | (*b*) Provide in the abstract an informative and balanced summary of what was done and what was found | p. 2; lines 30-57 |
| Introduction | | |  |
| Background/rationale | 2 | Explain the scientific background and rationale for the investigation being reported | p. 4; lines 67-153 |
| Objectives | 3 | State specific objectives, including any prespecified hypotheses | p. 7; lines 153-161 |
| Methods | | |  |
| Study design | 4 | Present key elements of study design early in the paper | p. 9; lines 191-206 |
| Setting | 5 | Describe the setting, locations, and relevant dates, including periods of recruitment, exposure, follow-up, and data collection | p. 7; lines 164-165 |
| Participants | 6 | (*a*) Give the eligibility criteria, and the sources and methods of selection of participants | p. 7; lines 165-178 |
| Variables | 7 | Clearly define all outcomes, exposures, predictors, potential confounders, and effect modifiers. Give diagnostic criteria, if applicable | p. 9; lines 208-237 |
| Data sources/ measurement | 8* | For each variable of interest, give sources of data and details of methods of assessment (measurement). Describe comparability of assessment methods if there is more than one group | p. 9; lines 208-237 |
| Bias | 9 | Describe any efforts to address potential sources of bias | p. 9; lines 199-201 |
| Study size | 10 | Explain how the study size was arrived at | - |
| Quantitative variables | 11 | Explain how quantitative variables were handled in the analyses. If applicable, describe which groupings were chosen and why | p. 10; lines 239-285 |
| Statistical methods | 12 | (*a*) Describe all statistical methods, including those used to control for confounding | p. 10; lines 239-285 |
|  |  | (*b*) Describe any methods used to examine subgroups and interactions | p. 10; lines 239-285 |
|  |  | (*c*) Explain how missing data were addressed | - |
|  |  | (*d*) If applicable, describe analytical methods taking account of sampling strategy | Not applicable |
|  |  | (*e*) Describe any sensitivity analyses | Not applicable |
| Results | | |  |
| Participants | 13* | (a) Report numbers of individuals at each stage of study—eg numbers potentially eligible, examined for eligibility, confirmed eligible, included in the study, completing follow-up, and analysed | p. 7; lines 165-178 |
|  |  | (b) Give reasons for non-participation at each stage | - |
|  |  | (c) Consider use of a flow diagram | - |
| Descriptive data | 14* | (a) Give characteristics of study participants (eg demographic, clinical, social) and information on exposures and potential confounders | Table 1; p. 8; lines 179-182 |
|  |  | (b) Indicate number of participants with missing data for each variable of interest | - |
| Outcome data | 15* | Report numbers of outcome events or summary measures | Table 1; p. 8; lines 179-182 |
| Main results | 16 | (*a*) Give unadjusted estimates and, if applicable, confounder-adjusted estimates and their precision (eg, 95% confidence interval). Make clear which confounders were adjusted for and why they were included | Table 2; p. 14; lines 322-324 |
|  |  | (*b*) Report category boundaries when continuous variables were categorized | - |
|  |  | (*c*) If relevant, consider translating estimates of relative risk into absolute risk for a meaningful time period | - |
| Other analyses | 17 | Report other analyses done—eg analyses of subgroups and interactions, and sensitivity analyses | p. 15; lines 346-363 |
| Discussion | | |  |
| Key results | 18 | Summarise key results with reference to study objectives | p. 17; lines 401-411 |
| Limitations | 19 | Discuss limitations of the study, taking into account sources of potential bias or imprecision. Discuss both direction and magnitude of any potential bias | p. 21; line 511-535 |
| Interpretation | 20 | Give a cautious overall interpretation of results considering objectives, limitations, multiplicity of analyses, results from similar studies, and other relevant evidence | p. 16; line 412-469 |
| Generalisability | 21 | Discuss the generalisability (external validity) of the study results | p. 20; lines 470-510; 511-513 |
| Other information | | |  |
| Funding | 22 | Give the source of funding and the role of the funders for the present study and, if applicable, for the original study on which the present article is based | Not applicable |

*Give information separately for exposed and unexposed groups.

**Note:** An Explanation and Elaboration article discusses each checklist item and gives methodological background and published examples of transparent reporting. The STROBE checklist is best used in conjunction with this article (freely available on the Web sites of PLoS Medicine at http://www.plosmedicine.org/, Annals of Internal Medicine at http://www.annals.org/, and Epidemiology at http://www.epidem.com/). Information on the STROBE Initiative is available at www.strobe-statement.org.
